# The 8th AJCC classification is inferior to a new neck stage based on intraparotid lymph node in parotid gland cancer

**DOI:** 10.1186/s12903-024-04346-y

**Published:** 2024-05-21

**Authors:** Xiaoxue Han, Changyu Yang, Xuexin Tan, Yuexiao Li

**Affiliations:** 1 Department of Oral and Maxillofacial Surgery, School of Stomatology, China Medical University, No.117 Nanjing North Street, Heping District, Shenyang, 110002 Liaoning P.R. China; 2 Department of Stomatology, The Fourth Affiliated Hospital of China Medical University, Shenyang, P.R. China

**Keywords:** Parotid cancer, Intraparotid lymph node, Extranodal extension, Lymph node burden, AJCC

## Abstract

**Background:**

Lymph node (LN) status is an important prognostic factor for parotid gland cancer (PGC). This study aimed to analyze the impact of extranodal extension (ENE) of intraparotid LN and LN metastasis burden on survival in PGC.

**Methods:**

Patients with surgically treated PGC and at least one metastatic cervical LN were retrospectively enrolled. Primary outcome variables were distant metastasis-free survival (DMFS), disease-specific survival (DSS), and overall survival (OS). The impact of ENE and LN metastasis burden was assessed using the Cox model.

**Results:**

A total of 292 patients were included. ENE in cervical or intraparotid LN was not associated with DMFS, DSS, or OS. Intraparotid LN metastasis had a significant impact on prognosis, and the presence of only one metastatic intraparotid LN offered an approximately 1.5-fold risk of distant metastasis. Prognostic models based on the number of positive LNs (1 vs. 2–3 vs. 4+) were superior to the AJCC N stage in terms of DMFS, DSS, and OS.

**Conclusions:**

ENE of cervical or intraparotid LN has a limited effect on the prognosis of PGC, and the number of positive LNs is better than the AJCC N stage in LN status evaluation.

## Introduction

Parotid gland cancer (PGC) accounts for almost 60–80% of all salivary gland malignancies [1]. Surgery is the mainstay of therapy, distant metastasis is the most common type of treatment failure. One of the significant prognostic factors is lymph node (LN) status, which is determined by the size and number of LNs, and extranodal extension (ENE), as explained by the AJCC neck staging system [2]. However, this official stage is formulated according to head and neck squamous cell carcinoma, and there are apparent differences in biological features between head and neck squamous cell carcinoma and PGC [3]. There have been discussions concerning the rationality of the official N stage in PGC [4, 5].

Some literature has confirmed the prognostic role of the number of metastatic LNs in PGC, and prognostic models based on LN burden have predicted superior survival when compared to the AJCC N stage [4, 6–9]. However, among these researches, the impact of ENE has shown conflicting results. Few studies have described that ENE poses little effect on survival in PGC [4, 5, 7, 9]; however, Lee et al. [8] reported that patients with ENE have an increased possibility of recurrence and death. Contrarily, the embryologic development of the parotid gland raises the possibility of intraparotid LNs, which can also be involved with metastasis. The role of the number and ENE of positive intraparotid LN has been rarely assessed [10].

Therefore, this study aimed to evaluate the impact of ENE of the cervical and intraparotid LNs on the survival of PGCs with metastatic LNs.

## Patients and methods

### Ethical consideration

This study was approved by China Medical University Institutional Research Committee, and informed consent was obtained from all subjects before initial research. All methods were performed in accordance with the relevant guidelines and regulations.

### Patient selection

The medical records of patients with PGC between January 2000 and December 2022 were retrospectively reviewed, and the inclusion criteria were as follows: the disease was primary and epithelial; primary site surgery and neck dissection were performed; at least 10 cervical LNs dissected in total and one was a positive LN. Patients without follow-up data were excluded. Information regarding the demography, pathology, treatment, and follow-up of the enrolled patients was extracted.

### Variable definition

All the pathological sections were reviewed by at least two head and neck pathologists. Tumor and neck stages were formulated according to the 8th AJCC system. The histological grade was classified as low, intermediate, or high according to the 5th version of the World Health Organization Classification for salivary gland tumors [10, 11]. Lymphovascular invasion (LVI) was defined as positive if there were cancer cells within the lymphatics. Perineural invasion (PNI) was considered positive if cancer cells had invaded a nerve [12]. ENE was defined as the presence of cancer cells outside the LN capsule and compared as none vs. ENE in intraparotid LN vs. ENE in cervical LN vs. ENE in both intraparotid and cervical LNs. LN size was defined as the largest diameter of metastatic cervical LNs. Margin was positive if there were cancerous cells present at the outer edge or margin of the removed tissue specimen [13].

Primary outcome variables were overall survival (OS), disease-specific survival (DSS), and distant metastasis-free survival (DMFS). OS was calculated from the date of surgery to the date of death or the last follow-up. DSS was calculated from the date of surgery to the date of cancer-caused death or the last follow-up. DMFS was calculated from the date of surgery to the date of the first detection of distant metastasis or the last follow-up.

### Treatment principle

At our cancer center, all patients underwent ultrasound and CT/MRI for PGC, with PET/CT occasionally employed for additional assessment of neck and distant metastases. Surgery at the primary site consisted of superficial and total parotidectomies, and neck dissection for PGC was selectively performed. Indications for lymphadenectomy included an advanced tumor stage, high histologic grade, and pathologically or clinically positive neck metastasis. Adjuvant radiotherapy was indicated in cases featuring LN metastasis, stage T3/4 tumors, high histologic grade, PNI, LVI, ENE, or positive margins. Furthermore, the consideration of adjuvant chemotherapy was inclined towards instances exhibiting ENE or positive margins. At least level I-IV LNs were dissected and level V was also excised when there was level II metastasis.

### Statistical analysis

The associations between clinicopathological variables and OS, DSS, and DMFS were first assessed using univariate analysis, and significant factors were further analyzed using the Cox model. Prognostic models were constructed according to different LN evaluation methods: in model 1, LN status was evaluated using the AJCC N stage; in model 2, LN status was assessed by the total number of metastatic LNs based on the results of binary recursive partitioning analysis. The impact of independent variables on survival is presented as hazard ratios (HR) and 95% confidence intervals (CI). All statistical analyses were performed using R 3.4.4, and statistical significance was set at *P* < 0.05.

## Results

### Baseline data

A total of 292 patients were included. There were 182 females and 110 males with a mean age of 52 ± 20 years. The clinical tumor stages encompassed T1, T2, T3, and T4 classifications in 24, 113, 91, and 64 patients, respectively. However, post-surgery, 10 patients were downstaged from T2 to T1, 6 patients were upstaged from T3 to T4, and 10 patients were downstaged from T4 to T3. The most common histological type was mucoepidermoid carcinoma, followed by myoepithelial carcinoma; the least frequent type was epithelial-myoepithelial carcinoma (Table 1). The histological grade was low, intermediate, and high in 66, 141, and 85 patients, respectively. PNI and LVI were observed in 52 and 43 patients, respectively. All the patients underwent total parotidectomy. Positive margins were observed in 15 patients.

**Table 1:** Histologic type distribution of parotid gland cancers

Cancer type	*N*
High grade (*n* = 85)	
Mucoepidermoid carcinoma	34
Duct carcinoma	19
Adenocarcinoma not otherwise specified	12
Spindle cell carcinoma	11
Large/small cell carcinoma	9
Intermediate grade (*n* = 141)	
Mucoepidermoid carcinoma	88
Myoepithelial carcinoma	35
Adenoid cystic carcinoma	18
Low grade (*n* = 66)	
Mucoepidermoid carcinoma	32
Acinic cell carcinoma	18
Pleomorphic low-grade adenocarcinoma	6
Basal cell carcinoma	6
Epithelial-myoepithelial carcinoma	4

Pathologic neck stage was N1, N2, and N3 in 162, 77, and 53 patients, respectively. The mean number of metastatic LNs was 2 ± 2 with a range from 1 to 15. The median size of metastatic LN was 2.5 cm with a range from 0.4 cm to 7.5 cm. ENE was noted in 39 patients. Intraparotid LN metastasis occurred in 156 patients, and the mean number of intraparotid metastatic LNs was 1 ± 1 with a range from 1 to 6. ENE was observed in 43 patients. The median ratio of positive to total LNs was 0.15 (range: 0.01-1.00). Adjuvant radiotherapy was administered to all the patients and 123 patients also received adjuvant chemotherapy.

During a mean follow-up duration of 79 ± 31 months, distant metastasis occurred in 60 patients and 114 patients died, among whom 90 deaths were caused by the cancer.

### Univariate analysis

**Fig. 1:**
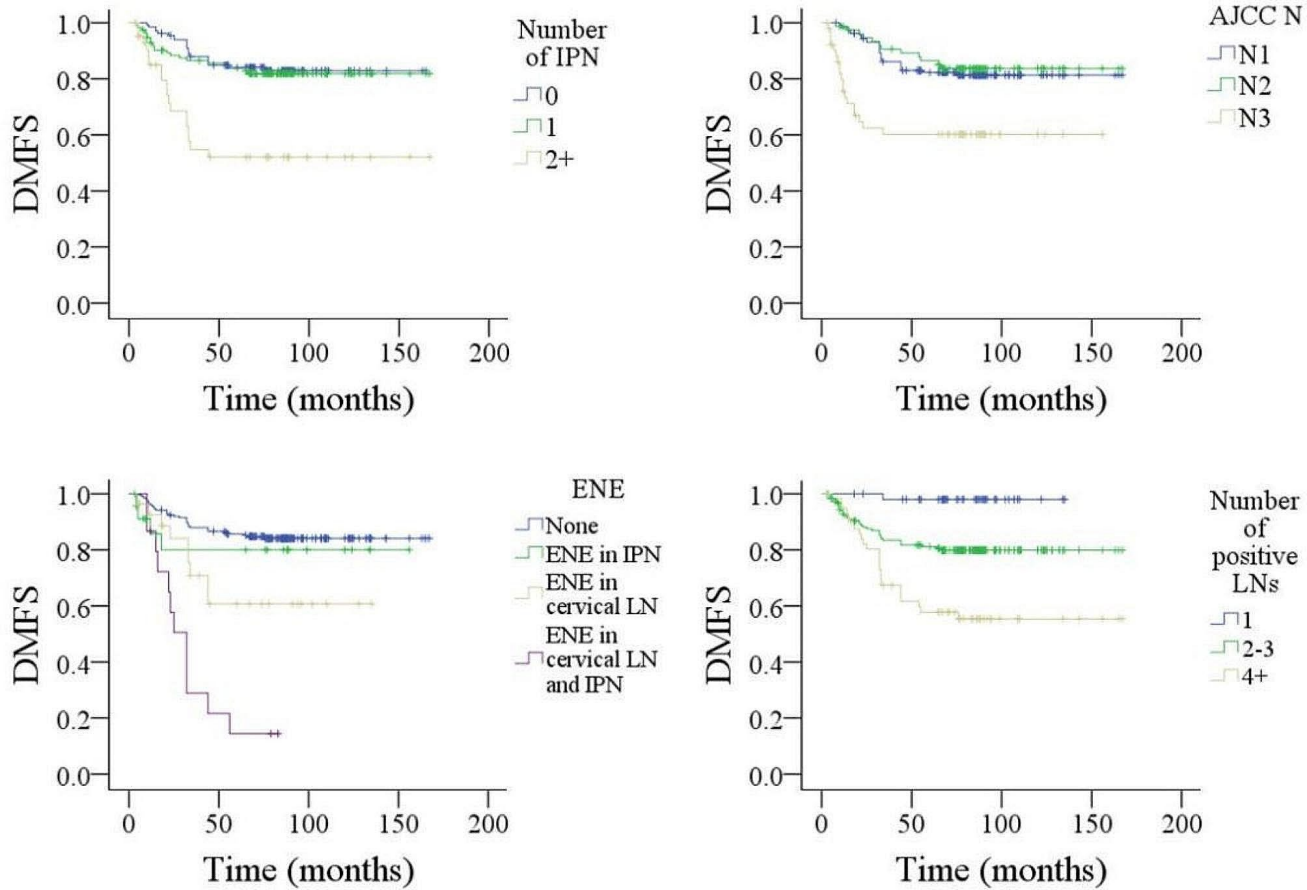
Comparison of distant metastasis free survival (DMFS) in patients with different features. **A** for number of metastatic intraparotid lymph node (IPN); **B** for AJCC N stage; **C** for extranodal extension (ENE); **D** for number of total positive lymph nodes (LNs)

**Fig. 2:**
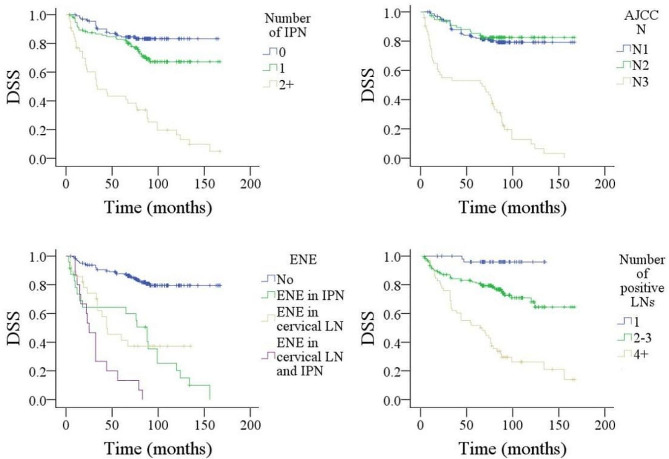
Comparison of disease specific survival (DSS) in patients with different features. **A** for number of metastatic intraparotid lymph node (IPN); **B** for AJCC N stage; **C** for extranodal extension (ENE); **D** for number of total positive lymph nodes (LNs)

**Fig. 3:**
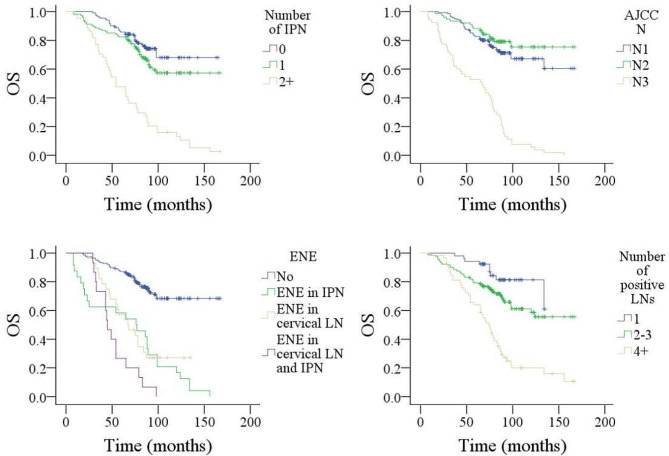
Comparison of overall survival (OS) in patients with different features. **A** for number of metastatic intraparotid lymph node (IPN); **B** for AJCC N stage; **C** for extranodal extension (ENE); **D** for number of total positive lymph nodes (LNs)

Factors such as tumor stage, histological grade, neck stage, positive margins, number of positive intraparotid LN, size of metastatic LN, ENE, and total number of positive LNs were significantly associated with DMFS, DSS, and OS (Figs. 1, 2 and 3, all *p* < 0.05). Age > 50 years was related to worse OS (*p* < 0.001). LVI predicted inferior DSS (*p* < 0.001) but not DMFS (*p* = 0.314) or OS (*p* = 0.652). Level IV/V metastasis indicated a higher possibility of distant metastasis (*p* < 0.001). Other variables did not impact DMFS, DSS, or OS (all *p* > 0.05) (Table 2).

**Table 2:** Univariate analysis of clinicopathologic variables in distant metastasis free survival (DMFS), disease specific survival (DSS), and overall survival (OS) in parotid gland cancer

Variable	DMFS	DSS	OS
Age (> 50 vs. ≤ 50)	0.432	0.116	< 0.001
Sex	0.836	0.518	0.732
Tumor stage	< 0.001	< 0.001	< 0.001
Perineural invasion	< 0.001	< 0.001	0.435
Lymphovascular invasion	0.314	< 0.001	0.652
Histologic grade	< 0.001	< 0.001	< 0.001
Neck stage	< 0.001	< 0.001	< 0.001
Positive margin	< 0.001	< 0.001	< 0.001
Adjuvant chemotherapy	0.537	0.366	0.475
Level involvement ( IV/V vs. I-III)	< 0.001	0.546	0.222
Number of positive intraparotid LN^	< 0.001	< 0.001	< 0.001
Number of total positive LN	< 0.001	< 0.001	< 0.001
ENE^#^	< 0.001	< 0.001	< 0.001
Size of metastatic LN	< 0.001	< 0.001	< 0.001
Ratio of positive to total LNs	0.143	0.632	0.474

^ LN: lymph node; # ENE: extranodal extension;

### Prognostic model for DMFS (Table 3)

**Table 3:** Multivariate analysis of clinicopathologic variables in distant metastasis free survival (DMFS) in parotid gland cancer

Variable	DMFS	
	*p*	HR[95%CI]
Multivariate Cox model 1		
Tumor stage		
T1-T2		ref
T3-T4	< 0.001	2.54[1.24–4.89]
Perineural invasion	0.103	2.06[0.74–6.17]
Histologic grade		
Low		ref
Intermediate	0.017	1.89[1.15–3.26]
High	< 0.001	3.56[1.63–8.04]
Neck stage		
N1		ref
N2	0.004	2.90[1.34–4.57]
N3	< 0.001	4.87[2.01–8.11]
Positive margin	< 0.001	5.21[1.98–12.35]
Number of positive intraparotid LN^		
0		ref
1	0.046	1.42[1.05–2.98]
2+	< 0.001	2.47[1.31–4.63]
Level involvement		
I-III		ref
IV/V	< 0.001	2.19[1.24–4.37]
Multivariate Cox model 2		
Tumor stage		
T1/2		ref
T3/4	< 0.001	2.43[1.18–4.68]
Perineural invasion	0.211	1.95[0.73–7.23]
Histologic grade		
Low		ref
Intermediate	0.035	1.67[1.10–3.15]
High	< 0.001	2.98[1.54–6.59]
ENE^&^		
None		ref
Intraparotid	0.327	1.99[0.76–2.68]
Cervical	0.136	2.31[0.82–3.90]
Intraparotid and cervical	0.111	2.34[0.87−5.00]
Positive margin	< 0.001	4.45[2.05–9.88]
Number of positive LN		
1		ref
2–3	0.004	2.52[1.27–5.03]
4+	< 0.001	4.34[2.45–13.17]
Level involvement		
I-III		ref
IV/V	< 0.001	2.73[1.37–5.24]
Size of metastatic LN		
~3 cm		ref
3.1 cm ∼ 6 cm	0.313	1.87[0.56–3.90]
6.1 cm~	0.207	3.03[0.66–7.12]

^ LN: lymph node

In Model 1 for DMFS, both neck stage and intraparotid LN metastasis emerged as independent variables of significance. Relative to the N1 stage, the N3 stage manifested the highest HR of 4.87 (95% CI: 2.01–8.11), while the N2 stage exhibited an HR of 2.90 (95% CI: 1.34–4.57). The presence of a solitary metastatic intraparotid LN was associated with a marginal elevation in the likelihood of distant metastasis (HR: 1.42; 95% CI: 1.05–2.98), whereas the presence of two or more positive LNs denoted an approximate 1.5-fold increase in the risk of distant metastasis (HR: 2.47; 95% CI: 1.31–4.63). In juxtaposition to low-grade tumors, both intermediate and high-grade malignancies were correlated with inferior DMFS outcomes, with HRs of 1.89 (95% CI: 1.15–3.26) and 3.56 (95% CI: 1.63–8.04), respectively. Additional independent prognostic factors encompassed T3/4 stage, positive surgical margin, and metastasis to level IV/V nodes. The model exhibited a C-index of 0.667 (95% CI: 0.662–0.673).

In Model 2 for DMFS, the presence of two or three metastatic LNs was linked to an elevated HR of 2.52 (95% CI: 1.27–5.03), while having four or more positive LNs constituted the highest risk category with an HR of 4.34 [95% CI: 2.45–13.17]. Contrastingly, both intermediate and high-grade malignancies exhibited poorer DMFS outcomes than low-grade tumors, demonstrating HRs of 1.67 (95% CI: 1.10–3.15) and 2.98 (95% CI: 1.54–6.59), respectively. Additional independent prognostic indicators comprised T3/4 stage, positive surgical margins, and metastasis to level IV/V nodes. In comparison to the absence of ENE, the presence of ENE in either the cervical or intraparotid LN did not confer a significantly increased risk of distant metastasis (all *p* > 0.05). This prognostic algorithm yielded a C-index of 0.680 (95% CI: 0.675–0.684).

### Prognostic model for DSS (Table 4)

**Table 4:** Multivariate analysis of clinicopathologic variables in disease specific survival (DSS) in parotid gland cancer

Variable	DSS	
	*p*	HR[95%CI]
Multivariate Cox model 1		
Tumor stage		
T1-T2		ref
T3-T4	< 0.001	2.87[1.35–6.34]
Perineural invasion	0.012	1.76[1.13–3.47]
Lymphovascular invasion	0.132	1.89[0.63–6.22]
Histologic grade		
Low		ref
Intermediate	0.013	1.67[1.11–3.08]
High	< 0.001	3.28[1.63–7.45]
Neck stage		
N1		ref
N2	< 0.001	1.87[1.18–3.46]
N3	< 0.001	3.75[1.54–8.42]
Positive margin	< 0.001	4.86[2.03–10.56]
Number of positive intraparotid LN^		
0		ref
1	0.218	1.78[0.78–2.47]
2+	< 0.001	2.00[1.32–4.69]
Multivariate Cox model 2		
Tumor stage		
T1/2		ref
T3/4	< 0.001	2.93[1.41–6.25]
Perineural invasion	0.006	1.43[1.07–2.99]
Lymphovascular invasion	0.204	1.67[0.78–5.34]
Histologic grade		
Low		ref
Intermediate	0.020	1.53[1.03–2.86]
High	< 0.001	3.12[1.56–7.42]
ENE^&^		
None		ref
Intraparotid	0.476	1.68[0.65–3.06]
Cervical	0.254	2.58[0.72–4.11]
Intraparotid and cervical	0.187	3.00[0.82–7.42]
Positive margin	< 0.001	4.99[2.22–12.43]
Number of positive LN		
1		ref
2–3	0.016	1.70[1.19–3.14]
4+	< 0.001	3.75[2.04–8.89]
Size of metastatic LN		
~3 cm		ref
3.1 cm ∼ 6 cm	0.472	1.90[0.67–3.37]
6.1 cm~	0.341	2.87[0.79–8.15]

^ LN: lymph node

In Model 1, neck stage and intraparotid LN metastasis emerged as independent prognostic determinants of DSS. Relative to the N1 stage, the N2 and N3 stages were associated with HRs of 1.87 (95% CI: 1.18–3.46) and 3.75 (95% CI: 1.54–8.42), respectively. While the presence of zero and one positive intraparotid LN yielded similar HRs, the presence of two or more positive LNs portended a markedly worse DSS outcome (HR: 2.00; 95% CI: 1.32–4.69). In contrast to low-grade tumors, both intermediate and high-grade malignancies exhibited inferior DSS, with HRs of 1.67 (95% CI: 1.11–3.08) and 3.28 (95% CI: 1.63–7.45), respectively. Other prognostic factors encompassed T3/4 stage, PNI, and positive surgical margins. The model demonstrated a C-index of 0.691 (95% CI: 0.688–0.698).

In Model 2 concerning DSS, compared with group of 0/1 metastatic LN, the presence of two or three positive LNs carried a HR of 1.70 (95% CI: 1.19–3.14), while the existence of four or more metastatic LNs indicated an almost 2.7-fold escalation in the risk of mortality from cancer. In contrast to tumors of low grade, both intermediate and high-grade malignancies correlated with diminished DSS, demonstrating HRs of 1.53 (95% CI: 1.03–2.86) and 3.12 (95% CI: 1.56–7.42), respectively. Additional independent variables encompassed the T3/4 stage, PNI, and positive margins. The presence of ENE in either the cervical or intraparotid LN did not exhibit a reduction in DSS compared to cases lacking ENE (all *p* > 0.05). This prognostic framework garnered a C-index of 0.695 (95% CI: 0.692-0.700).

### Prognostic model for OS (Table 5)

**Table 5:** Multivariate analysis of clinicopathologic variables in overall survival (OS) in parotid gland cancer

Variable	OS	
	*p*	HR[95%CI]
Multivariate Cox model 1		
Age (> 50 vs. ≤ 50)	< 0.001	1.36[1.13–1.89]
Tumor stage		
T1-T2		ref
T3-T4	< 0.001	2.65[1.29–6.20]
Histologic grade		
Low		ref
Intermediate	0.009	1.74[1.12–2.99]
High	< 0.001	3.34[1.46–6.68]
Neck stage		
N1		ref
N2	< 0.001	1.94[1.20–3.56]
N3	< 0.001	3.66[1.36–8.57]
Positive margin	< 0.001	4.90[2.21–11.80]
Number of positive intraparotid LN^		
0		ref
1	0.309	1.80[0.53–4.10]
2+	< 0.001	2.16[1.29–7.38]
Multivariate Cox model 2		
Age (> 50 vs. ≤ 50)	< 0.001	1.35[1.12−2.00]
Tumor stage		
T1/2		ref
T3/4	< 0.001	2.52[1.25–6.06]
Histologic grade		
Low		ref
Intermediate	0.010	1.69[1.21–2.87]
High	< 0.001	3.53[1.29–7.17]
ENE^&^		
None		ref
Intraparotid	0.645	2.14[0.63–3.07]
Cervical	0.444	2.59[0.70–4.43]
Intraparotid and cervical	0.439	3.31[0.73–6.27]
Positive margin	< 0.001	4.84[2.17–10.74]
Number of positive LN		
1		ref
2–3	0.023	1.55[1.15–3.02]
4+	< 0.001	3.98[2.11–9.10]
Size of metastatic LN		
~3 cm		ref
3.1 cm ∼ 6 cm	0.636	1.67[0.42–3.16]
6.1 cm~	0.290	2.83[0.64–6.78]

^ LN: lymph node

In Model 1, the neck stage and the presence of two or more metastatic intraparotid LNs emerged as independent risk determinants for OS. Relative to the absence of intraparotid LN metastasis, the existence of a single metastatic intraparotid LN had minimal impact on survival (HR: 1.80; 95% CI: 0.53–4.10). In contrast to low-grade tumors, both intermediate and high-grade malignancies were associated with reduced OS, with HRs of 1.74 (95% CI: 1.12–2.99) and 3.34 (95% CI: 1.46–6.68), respectively. Additional prognostic factors encompassed an age exceeding 50 years, T3/4 stage, and positive surgical margins. The model exhibited a C-index of 0.678 (95% CI: 0.673–0.683).

In Model 2 for OS, the presence of two or three positive LNs was associated with a HR of 1.55 (95% CI:1.15–3.02), while the presence of four or more metastatic LNs carried an HR of 3.98 (95% CI: 2.11–9.10). When juxtaposed with low-grade tumors, both intermediate and high-grade malignancies were linked to diminished OS, showcasing HRs of 1.69 (95% CI: 1.21–2.87) and 3.53 (95% CI: 1.29–7.17), respectively. Other notable factors encompassed an age surpassing 50 years, T3/4 stage, and positive surgical margins. Relative to cases lacking ENE, the presence of ENE in either the cervical or intraparotid LN did not correlate with an additional risk of mortality (all *p* > 0.05). This prognostic model yielded a C-index of 0.681 (95% CI: 0.677–0.687).

## Discussion

Our most important finding was that ENE had a limited effect on prognosis in PGC. However, the intraparotid LN metastasis burden was significantly associated with survival, and LN status evaluation based on the number of positive LNs provided superior survival clarification compared to the AJCC N stage. Our study offers an alternative LN assessment method with better screening of high-risk patients, although further validation is required.

ENE is an important feature of LN status, indicating poor prognosis and the necessity of adjuvant therapy [14]. It has been considered in the newest version of the AJCC system; however, its role in salivary gland cancer has rarely been analyzed and remains controversial. Hsieh et al. [4] enrolled 114 patients with pN + salivary gland cancer; ENE developed in 58 patients and did not affect regional control, locoregional control, DMFS, disease-free survival, or OS. Lombardi et al. [5] reported ENE in 61.5% of 91 patients with major salivary gland cancer, of whom ENE of cervical LN was observed in 59.1% and ENE of intraparotid LN was observed in 41.9%; however, the Kaplan-Meier survival curves showed no statistically significant difference between ENE + and ENE- patients. Aro et al. [9] analyzed 4520 patients with salivary gland cancer via a public database and noted that although ENE was associated with a nearly 2-fold increased risk of death in univariate analysis, the association was no longer available in multivariate analysis. These three studies did not clarify the impact of ENE originating from the cervical or intraparotid LN. Fang et al. [8] recently reported that ENE developed in 23.1% of metastatic intraparotid LNs, and its presence did not affect recurrence-free survival or OS in 453 patients with PGC. However, the authors failed to find an association between ENE and distant metastasis, the risk of which was significantly increased in solid cancers by ENE. Our previous study assessed the predictors of DMFS in 232 patients with adenoid cystic carcinoma and reported that ENE in neither the cervical nor the intraparotid LN affected DMFS [7]. The current study is a further supplement, confirming that ENE did not compromise survival, irrespective of LN location. However, Lee et al. [6] argued that ENE could significantly decrease OS, DSS, and disease-free survival based on their study including 172 patients with salivary gland cancer. This difference might be explained by the fact that only intermediate and high histologic-grade diseases were included in this study. Unfortunately, no similar studies are available for comparison, and this issue requires further discussion.

The survival significance of intraparotid LN is well recognized, and the presence of intraparotid LN metastasis is related to worse prognosis [15, 16]; however, few authors have analyzed the role of intraparotid LN metastasis burden in PGC. Feng et al. [10] might have been the first to address this topic. In their research, the 10-year local control rate was 94% for patients without intraparotid LN metastasis, 56% for patients with metastasis in no more than two metastatic intraparotid LNs, and 22% for patients with metastasis in more than two positive intraparotid LNs; this difference was significant in both univariate and multivariate analyses. In another study by Fang et al. [8], the authors assessed the impact of intraparotid LN metastasis in detail and divided the patients into three groups based on 0 vs. 1 vs. 2 + metastatic intraparotid LNs; multivariate analysis showed that the group with one metastatic LN had comparable recurrence-free survival and OS compared to the group with no intraparotid LN metastasis. However, the presence of two or more positive LNs predicted significantly decreased disease control and increased death risk. Our previous study showed that intraparotid LN metastasis was significantly associated with inferior prognosis; however, the impact was not apparent until there were at least two metastatic intraparotid LNs in parotid adenoid cystic carcinoma [7]. These three studies combined with ours suggest that the impact of the intraparotid LN metastasis burden should be emphasized in the evaluation of LN status.

A good LN stage should be simple for clinical application and accurate for survival stratification; however, the AJCC N stage takes the size, location, number, and ENE of LN into consideration [17]. This is not sufficient for PGC. Contralateral neck LN metastasis was extremely rare in PGC and only accounted for 0.3% in a large-scale study [9]; contrarily, the effect of intraparotid LN was ignored. Therefore, an alternative N stage is required. Aro et al. [9] first noted that an increasing number of metastatic LNs was found to be strongly associated with OS without a plateau; the risk increased more rapidly up to four LNs and was more gradual for additional LNs > four, and its effect was determined by the number of metastatic LNs other than LN size, ENE, and lower LN involvement. The proposed N stage (0 vs. 1–2 vs. 3–21 vs. 22 + metastatic LNs) exhibited a greater C-index than the AJCC N system. Subsequent researchers suggested other N stages based on the LN metastasis burden, and all of them had better survival prediction than the AJCC N stage [4–6, 8]. This finding was also supported by our study, more importantly, distant metastasis, which was a main cause of treatment failure in PGC [18], was chosen as a primary outcome variable in this study, it first reported the predictive role of LN stage according to the number of metastatic LNs.

Other well-known prognostic factors include the ratio of positive to total LNs, level IV/V involvement, and the logarithmic ratio of positive lymph nodes. Lower LN metastasis is a risk indicator of distant metastasis and adjuvant chemotherapy [19]; however, after adjusting for the number of positive LNs, it was no longer associated with DMFS. Some studies commented that the ratio and logarithmic ratio of positive to total LNs acted as independent prognostic factors [20, 21]; however, this was not accurate. For example, the presence of two positive LNs could be incorporated into a high-risk group if only ten LNs were dissected, or into a low-risk group if 50 LNs were dissected. The survival was determined by a number other than the ratio. Moreover, it is difficult to calculate the logarithmic ratio. Prior research substantiated the pivotal role of pathologic grade as a significant prognostic determinant [5, 8], a notion further corroborated by our investigation. In comparison to low-grade tumors, both intermediate and high-grade malignancies portended elevated risks of distant metastasis and mortality. The efficacy of adjuvant chemotherapy in salivary gland cancer has been subject to considerable scrutiny, with enhanced outcomes solely documented in the subset of squamous cell carcinoma within salivary gland malignancies. Regrettably, despite extensive inquiry, a definitive association between chemotherapy and prognosis has eluded the majority of researchers, including ourselves [22–24].

It is imperative to acknowledge the limitations of the present study. Primarily, there exists an inherent selective bias stemming from the retrospective design of the study. Additionally, the data were sourced from a solitary center, and our sample size was somewhat modest; therefore, corroborative evidence from a comprehensive multicenter study is essential.

In summary, ENE of the cervical or intraparotid LN had limited effect on the prognosis of PGC, and LN status evaluation based on the number of positive LNs provided superior survival clarification than the AJCC N stage.

## Data Availability

All data generated or analyzed during this study are included in this published article. And the primary data could be achieved from the corresponding author.
